# The Role of Allogeneic Stem Cell Transplantation in Relapsed/Refractory Hodgkin's Lymphoma Patients

**DOI:** 10.1155/2011/974658

**Published:** 2010-10-26

**Authors:** Evgeny Klyuchnikov, Ulrike Bacher, Nicolaus Kröger, Ilya Kazantsev, Tatjana Zabelina, Francis Ayuk, Axel Rolf Zander

**Affiliations:** ^1^Interdisciplinary Clinic for Stem Cell Transplantation, University Cancer Center Hamburg (UCCH), Martinistr. 52, 20246 Hamburg, Germany; ^2^Clinic for Stem Cell Transplantation, St. Petersburg State, Pavlov's Medical University, St. Petersburg 197022, Russia

## Abstract

Despite the favorable prognosis of most patients with Hodgkin's Lymphoma (HL), 15–20% of patients remain refractory to chemoradiotherapy, and 20–40% experience relapses following autologous stem cell transplantation (SCT) being used as salvage approach in this situation. Long-term survival of only 20% was reported for patients who failed this option. As some authors suggested the presence of a graft versus HL effect, allogeneic SCT was introduced as a further option. Myeloablative strategies were reported to be able to achieve cure in some younger patients, but high nonrelapse mortality remains a problem. Reduced intensity conditioning, in turn, was found to be associated with high posttransplant relapse rates. As there is currently no standard in the management of HL patients who failed autologous SCT, we here review the literature on allogeneic stem cell transplantation in HL patients with a special focus on the outcomes and risk factors being reported in the largest studies.

## 1. Introduction

During the last decades, survival of patients with Hodgkin's Lymphoma (HL) has substantially improved. To date, 80%–85% of patients achieve stable remissions [1]. Even in advanced stages (IIB with large mediastinal tumors and all stages III-IV according to the Ann Arbor classification), more than 80% of patients experience long-term tumor-free survival. Nevertheless, 15%–20% of HL patients remain refractory or develop relapse/progression after an initial chemoradiotherapy. For these patients, high-dose chemotherapy followed by autologous stem cell support (HD-SCT) represents a more efficient strategy when compared to conventional chemoradiotherapy [2, 3]. Nevertheless, a total of 20–40% patients undergoing chemoradiotherapy and/or HD-SCT develop relapses during a followup period of 7 years after treatment [4–9]. Median survival following HD-SCT failure was reported to range from 6 to 84 months [8–10]. 

Salvage strategies to improve outcomes for this group of patients include use of chemotherapy (e.g., gemcitabine-based regimens) [11], a second HD-SCT [12], and allogeneic stem cell transplantation (SCT). Allogeneic SCT for relapsed/refractory HL patients, first reported in the 1980's [13, 14], was effective to allow disease control in some of those, but on the other hand was associated with high transplant-related mortality (TRM) rates [15, 16]. Therefore, and based on the assumption of a possible allogeneic graft *versus* lymphoma (GvL) effect, it was suggested to introduce reduced intensity conditioning [17, 18]. Nevertheless, as the existence of a GvL effect in patients with HL remains controversial, it seems difficult to estimate the role of allogeneic SCT for relapsed/refractory HL following HD-SCT. Moreover, given the rare occurrence of relapsed/refractory HL after failure of HD-SCT, most studies focusing on allogeneic SCT were based on limited case series. This paper summarizes the most relevant studies on the use of allogeneic SCT in relapsed/refractory HL patients.

## 2. Introduction of Allogeneic SCT in HL

The first systematic evaluations of allogeneic SCT in relapsed HL were published in the 1990's [16, 19]. For instance, Gajewski et al. [16] analyzed outcomes for 100 patients with relapsed/refractory HL. Median age of patients was 24 years (range, 12–44). The majority of patients experienced advanced disease, and only eleven patients were in remission at the time of transplantation. The myeloablative regimens (MAC) were based on combinations of busulfan (16 mg/kg) and cyclophosphamide (200 mg/kg) with or without etoposide (20–60 mg/kg); or TBI (12 Gy) with cyclophosphamide. The results of the study were disappointing: owing to high relapse (65%) and nonrelapse mortality rates (61%), 3-year overall (OS) and disease free survival (DFS) rates were only 21% and 15%, respectively. Similar to previous reports, the authors observed a lower relapse risk in patients who developed acute and chronic GVHD, albeit not significant [15, 16, 19–21].

Other studies from the 1990's suggested that application of allogeneic strategies in patients with relapsed/refractory HL was limited by high NRM rates varying from 40% to 60% [19, 20]. According to such poor results, it was critically discussed whether myeloablative allogeneic SCT had a therapeutic potential for this cohort of patients. 

On the other hand, Cooney et al. published an interesting report on ten relapsed/refractory HL patients (median age 35 years; range, 21–49) who underwent myeloablative allogeneic SCT following the BEAM (BCNU, etoposide, cytarabine, and melphalan) conditioning regimen usually being reserved for autologous SCT. All patients had failed to previous HD-SCT. Six patients had chemosensitive disease with complete or partial remission at the time of allogeneic SCT, while four were refractory to previous chemotherapy. The authors observed no treatment-related deaths at 100 days posttransplant, and response was seen in all ten patients from the study (CR, *n* = 8; PR, *n* = 2). In five patients, response to allogeneic SCT was associated with the appearance of chronic GVHD. At the time of the report, nine patients were alive, of those seven in continuous complete remission (1 to 21 months from allogeneic SCT), while only one patient had died due to progression [22]. Although this series suggests that myeloablative conditioning is an optimal approach for relapsed/refractory HL patients, the study was limited by the number of enrolled patients and by short-term median followup of 12 months. Nevertheless, this report might serve as an example that some myeloablative strategies seem to be able to reduce NRM as compared to other intensive conditioning strategies (e.g., busulfan/cyclophosphamide, BuCy).

## 3. Reduced Intensity Conditioning

After basic research studies, reduced-intensity conditioning (RIC) followed by allogeneic SCT has been introduced for different hematologic entities, and has been found beneficial, for example, for patients who are considered poor candidates for myeloablative regimens [17, 23, 24]. Based on the observation of the high NRM in relapsed/refractory HL patients probably due to the toxicity load of previous treatments, RIC regimens were as well suggested in the setting of HL. Indeed, the introduction of allogeneic SCT following RIC for relapsed/refractory HL patients resulted in a decreased cumulative incidence of NRM ranging from 11% to 13%. Nevertheless, survival outcomes were relatively unchanged, as approximately 50% of all patients undergoing allogeneic SCT after RIC relapsed (Table 1) [25–27].

Robinson et al. retrospectively analyzed 285 adult relapsed/refractory HL patients undergoing different RIC regimens (so far the largest study on the use of RIC in this setting). The majority of patients (253/285, 89%) were <45 years and experienced chemosensitive disease; most had a history of prior HD-SCT. RIC regimens consisted of fludarabine/melphalan (FluMel), busulfan/fludarabine (BuFlu), and fludarabine/cyclophosphamide (FluCy) alone or with thiotepa. Transplantations were performed either from related (HLA matched, *n* = 172; mismatched, *n* = 8) or unrelated donors (HLA matched, *n* = 94; mismatched, *n* = 11). The early and cumulative 3-year TRM rates were relatively low (11% and 21%, resp.). Nevertheless, the 5-year progression rate in this study was increasing up to 59%. Thus, 3-year OS and progression free survival (PFS) were 29% and 25% only. Acute (grade II-IV) and chronic GvHD were seen in 34% and 38% of patients, respectively. The authors observed a trend to lower relapse rate for patients with GvHD, but there was no impact on PFS or OS [25]. Comparable results were demonstrated by previous studies on use of RIC regimens in HL patients: though the TRM rate was lower, overall and disease-free survival did not exceed 50% (Table 1). 

Recently, Sarina et al. published the results of a retrospective multicenter study on 185 relapsed/refractory HL patients. In this study, outcomes were correlated with donor availability. A total of 122 of patients (66%) had a suitable donor. The patients from the “donor group” experienced improved 2-year OS and PFS as compared with those from the “no donor group” (OS: 66% versus 42%, and PFS: 39% versus 14%, *P* < .001). The 2-year NRM rate for the transplanted patients was 13% [28]. 

Thus, although reduced intensity regimens provide improved survival outcomes and reduced NRM rates as compared to myeloablative strategies in HL patients, increased relapse or progression rates remain unsolved problems.

## 4. Arguments for a Graft versus Hodgkin's Lymphoma Effect

In some hematologic entities, the existence of a graft *versus* malignancy effect has been proven without doubts. For instance, in chronic myeloid leukemia (CML), or multiple myeloma, allogeneic SCT as well as donor lymphocytes infusions (DLIs) being applied due to posttransplant disease recurrence were significantly associated with response [35–37]. Also in some lymphoproliferative entities, for example, chronic lymphocyte leukemia (CLL), or follicular lymphoma (FL), such immunologic effect has been clearly demonstrated [38, 39]. Whether such effect exists as well in HL, has been controversially discussed [16, 26, 27, 40], but many arguments support this assumption [25, 41]. Alvarez et al. showed a trend to improved PFS when they compared HL patients who developed extensive chronic GvHD and those who did not (71% versus 44%, *P* = .07) [32]. Therefore, a beneficial effect of DLIs was suggested—for example, due to the low proliferation rate of HL—even in case no lymphoma reduction could be achieved by cytotoxic chemo- or radiotherapy. Beyond that, some larger studies on allogeneic SCT in patients with relapsed/refractory HL documented lower relapse rates in case of chronic GvHD [25, 34]. In conclusion, there is some evidence of a GvL effect following either allogeneic SCT or DLIs in HL [19–21, 25, 30, 32–34]. 

Considering the limitations to confirm a graft *versus* malignancy effect in patients with HL, as compared, for instance, to those with chronic myeloproliferative disorders, the role of the microenvironment seems to be essential in this specific setting. Several reports have described different intercellular interactions between HRS and nonmalignant cells, which can lead to immunologic escape [42]. Numerous mechanisms are being discussed: (i) production of immunosuppressive cytokines (e.g., IL-10, TGF*β*, galectin 1, or prostaglandin E2) by Hodgkin Reed Sternberg cells (HRS) [42–46]; (ii) expression of the Fas-Ligand (CD95L) [45]; (iii) downregulation of HLA class I antigens; (iv) expression of HLA-G antigens, which play an important role in the inhibition of the NK-cell response [47]; (v) expression of PD-1 (programmed death 1) protein, a negative regulator on the immune response of T cells and (by it's ligand) on HRS cells [48]. Such phenomenons were found to have a negative impact on survival [48, 49]. Also, Steidl et al. showed a strong association of increased numbers of tumor-associated macrophages (CD68^+^) with shortened survival in patients with classic HL [50]. As follows, novel “targeted” approaches, such as CD68^+^ cell depletion or use of PD-1/PD-1L binding antibodies, might be further evaluated in the setting of HL [51].

## 5. Reduced Intensity versus Myeloablative: Is It Possible to Define an Optimal Conditioning Strategy?

As mentioned above, the application of RIC considerably reduced the incidence of NRM even for patients with poor prognostic features at the time of allografting. However, the high relapse rates following allogeneic RIC-SCT remain a problem. On the other hand, high relapse rates were as well reported in MAC studies focusing on relapsed/refractory HL patients who had shown failure to autologous SCT (Table 1). 

The largest comparison between MAC and RIC regimens was performed by Sureda et al. who assessed outcomes for 168 relapsed/refractory HL patients receiving allografts (mostly from sibling donors). The majority of patients in both cohorts were ≤35 years, experienced stage III-IV HL, had received ≥3 lines of therapy before allogeneic SCT, and a history of HD-SCT (RIC: 62%, MAC: 41%). More than 50% of patients from both cohorts had chemoresistant disease. MAC regimens were based on combinations of cyclophosphamide with high-dose TBI (≥8 Gy) or busulfan (cum. 16 mg/kg). The RIC regimens included BEAM (BCNU, etoposide, cytarabine, and melphalan) or were based on fludarabine combined with an alkylating agent or low-dose TBI (2–4 Gy). Though the early cumulative incidence of acute GvHD was higher in the MAC group (53% versus 44% for RIC patients, *P* = .05), the incidence of chronic GvHD was similar in both groups (MAC: 38%; RIC: 40%). Although the patients from the RIC group were more heavily pretreated than those from the MAC group, the authors observed a lower cumulative NRM rate in the RIC patients (24% versus 48%, resp., *P* = .003). The 5-year rate of progression was significantly higher in the RIC when compared to the MAC cohort (58% versus 32%, resp.; *P* = .04). After a median followup of 75 months, 25% of the patients were alive (27% in the RIC and 23% in the MAC cohorts, resp.). The 5-year OS (MAC: 22%; RIC: 28%, *P* = .06) and PFS (MAC: 20%; RIC: 18%; *P* = .6) did not differ significantly [34]. Thus, RIC regimens are supposed to improve the long-term OS, but seem to have limited impact on disease progression as compared with MAC regimens. These results were in accordance to other studies. Anderlini et al. compared two fludarabine-based conditioning regimens (FluMel versus FluCy) in relapsed/refractory HL patients. The authors demonstrated improved OS and a trend to better PFS at 18 months post-allograft after the fludarabine/melphalan combination [26].

Sureda et al. showed no suitable evidence of a GvL effect [34]. These difficulties to clearly confirm a graft *versus* HL effect might be due to the high proportions of advanced and chemoresistant disease in the allotransplant setting, as the development of GvL is more feasible in case of a lower tumor burden. In this setting, the performance of HD-SCT being followed by allogeneic SCT allows to reduce the tumor burden prior to the development of a possible graft *versus* HL effect [52]. 

Therefore, it remains difficult to draw final conclusions concerning the most appropriate intensity of conditioning for relapsed/refractory HL patients who failed to achieve cure by high dose chemotherapy and autologous SCT. There is no doubt that the use of RIC improves OS in selected patients, but is linked to high relapse rates.

## 6. Outcomes According to Different Stem Cell Sources and Donor Types

Aiming to evaluate whether definite stem cell source would provide an immunologic conflict being relevant for a graft *versus* HL effect, Burroughs et al. evaluated 90 relapsed/refractory HL patients receiving grafts from *different donor types*. A total of 83 patients (92%) had failed to prior autologous HD- or syngeneic (allogeneic) SCT. The preparative regimens consisted of TBI (2 Gy) either alone or combined with fludarabine. For haploidentical SCT, the authors used a fludarabine/cyclophosphamide regimen combined with TBI (2 Gy). There were no cases of graft rejection. The 2-year OS was ranging from 53% to 58% in the different groups, without any significant differences. Strikingly, the early and 2-year NRM rates were lowest among unrelated and haploidentical graft recipients. The GvHD rates did not differ with respect to the different donor types. However, the authors reported on a decreased incidence of relapse in patients receiving allografts from haploidentical donors, which was suggestive for a graft versus HL effect [30]. Additionally, better results were observed in patients from the haploidentical cohort, who received cyclophosphamide as part of the conditioning. It was discussed, whether the drug is able to impair the T-regulatory cells of the microenvironment of Hodgkin/Reed-Sternberg (H/RS) cells [53–55].

Devetten et al. published the largest report on outcomes for 143 relapsed/refractory HL patients, all of whom received allografts from *unrelated* donors (HLA-matched, *n* = 110, 77%; mismatched, *n* = 33, 23%). A total of 127 patients (89%) had been previously autografted. The main conditioning regimens in this study were as follows: melphalan (Mel ≤ 150 mg/m^2^; *n* = 50); busulfan (Bu ≤ 9 mg/kg; *n* = 36); TBI (200 cGy; *n* = 25); fludarabine/cyclophosphamide (FluCy; *n* = 23). There was a low early TRM rate of 15%, but at two years it had increased up to 33%. Thus, the cumulative TRM rate in this study was higher than that reported by Robinson et al. (21% at 3 years posttransplant), most probably due to increased GvHD rates. Although the relapse rate was moderately lower in the study from Devetten et al. (47% at 2 years versus 59% at 5 years), the PFS was similar in both studies (Table 1) [25, 29].

Majhail et al. compared safety and efficacy of RIC-SCT using either unrelated umbilical *cord blood* (UCB, *n* = 9) *or matched sibling donors* (*n* = 21). Fourteen patients (67%) had a history of previous HD-SCT. The conditioning regimens were based on busulfan/fludarabine (BuFlu; *n* = 8) or TBI/cyclophosphamide (*n* = 13). There was no difference between the two groups with regards to 2-year PFS, early TRM, and the incidence of acute or chronic GvHD [56]. 

In conclusion, donor type and stem cell source seem to have no significant influence on posttransplant outcomes in HL patients [29, 30, 56].

## 7. Outcomes of Allogeneic SCT in Pediatric and Adolescent Patients Who Failed to HD-SCT

HD-SCT is increasingly used as salvage therapy also in pediatric patients with poor-risk HL [57, 58], although there are no randomized studies demonstrating improved outcomes for this approach as compared with conventional chemoradiotherapy. Information regarding the role of allogeneic SCT for HL in the pediatric population is very limited. Children undergoing allogeneic SCT have been occasionally included in series of adult patients [16, 21, 34]. Series focusing exclusively on pediatric patients were limited to fewer than 10 patients [40].

Claviez et al. published the largest series in pediatric or adolescent patients receiving allogeneic SCT (*n* = 91). A total of 40 patients (44%) failed to prior HD-SCT. Forty patients (44%) received myeloablative conditioning, in most cases based on busulfan/cyclophosphamide (BuCy) and TBI alone, or combined with etoposide or cytarabine. RIC regimens were performed in 51 patients (56%) and consisted of combinations of fludarabine with either melphalan (FluMel), busulfan (BuFlu), cyclophosphamide (FluCy), thiotepa, or low-dose TBI. The relapse rate was 34% in the MAC and 54% in the RIC cohort (*P* = .01). However, this had to be seen with regard to the different risk profiles, as patients with RIC had longer intervals between diagnosis and allogeneic SCT, had failed more lines of chemotherapy, including HD-SCT, and were significantly older than patients who underwent myeloablative conditioning. Primary graft failures occurred in five of 91 patients (6%), but in four cases stable engraftment was achieved after a second allogeneic SCT following RIC conditioning. A total of 21 patients (23%) died due to transplant-related causes. The frequencies of grade II-IV acute and chronic GvHD were 25% and 36%, respectively. Although in the early posttransplant period there was no difference in the relapse risk between the MAC and RIC cohorts, it became apparent that the relapse risk had significantly been increasing in patients who received RIC starting 9 months following transplantation. In addition, these patients had a significantly lower 3-year PFS [31]. 

Thus, more vigorous (not necessarily fully myeloablative) conditioning might represent a viable option to reduce the high relapse rates being seen in children and adolescents who tolerate aggressive chemotherapy as part of the conditioning regimen much better than older patients.

## 8. T-Cell Depletion

*In vivo* eradication of T cells during conditioning before allogeneic SCT was found to decrease the probability of GvHD [59, 60]. In case of HL, some T-cell depleting agents (e.g., monoclonal antibody alemtuzumab) might directly affect the tumor through the eradication of T cells, creating the tumor-supporting microenvironment [61]. Based on these assumptions, Peggs et al. investigated the role of *in vivo* T depletion in 67 relapsed/refractory HL patients most of whom had previously undergone HD-SCT. They used a fludarabine/melphalan (FluMel) regimen either with (*n* = 31) or without (*n* = 36) alemtuzumab (targeting CD52 positive cells). The cumulative TRM rate at two years was 7% in the alemtuzumab cohort and 29% in the “FluMel only” group [33]. It is difficult to determine whether either strategy had a significant influence on the relapse risk. Nevertheless, both the 3-year cumulative incidence of relapse (54% versus 44%) and 3-year PFS (43% versus 25%) were higher in patients with the FluMel/alemtuzumab regimen. This might have been associated to use of DLIs in the relapsed patients. Further, the authors confirmed the observation, that the inclusion of monoclonal antibodies (e.g., alemtuzumab or rituximab) into the preparative regimen significantly reduced both acute and chronic GvHD [62, 63]. In addition, there seemed to be a trend to longer duration of the responses in the alemtuzumab cohort (median, 33 months versus <12 months). Thus, the use of *in vivo* T-cell depletion with alemtuzumab did not seem to inhibit the GvL effect. In contrary, alemtuzumab might have a direct effect on the tumor thereby improving survival rates [33].

## 9. Alternative Strategies

For those patients who experience relapse of HL after allogeneic SCT, or who are no candidates for allogeneic SCT, alternative therapeutic approaches might be applied. These include the use of conventional or novel chemotherapeutic compounds (e.g., gemcitabine, pentostatin) [64–71], monoclonal antibody-based approaches (e.g., anti-CD20, anti-CD30, or radiolabeled antibodies) [72–76], use of histone deacetylase inhibitors (e.g., vorinostat) [77, 78], immunomodulatory drugs (e.g., thalidomide and lenalidomide) [79, 80], or their combinations [81, 82]. In addition, a pivotal trial with brentuximab—a novel agent consisting of an anti-CD30 monoclonal antibody and monomethyl auristatin E, an antimitotic agent—was recently completed [83]. The overall response (OR) rate in the cited studies varied from 25% to 76%. The most effective compounds were represented by gemcitabine (OR: 22%–76%), vinca alkaloids (OR: 46%–59%), and immunomodulatory drugs (OR: 33%–50%). Despite the relatively high response rate in some cases, responses were not stable (median duration 6 months). Nevertheless, Bartlett et al. reported on maximal progression free survival of 58 months after use of gemcitabine-based regimens (gemcitabine, vinorelbine, and pegylated liposomal doxorubicin) [65]. 

In conclusion, nowadays there is no alternative to allogeneic SCT strategy that allows to achieve long-time survival in relapsed/refractory HL patients. Therefore, allogeneic SCT most probably will continue to play an important role in this setting.

## 10. Conclusions

Although HL can be cured in the majority of cases with conventional chemoradiotherapy as first-line and HD-SCT as second-line strategies, there are no standard options for patients relapsing following autografting. Although there is no doubt that allogeneic SCT is an important and in some cases even curative strategy for relapsed/refractory HL patients, its role in this specific setting remains to be more clearly defined. First, transplantation rates are limited, and so is the number of large allotransplant studies focusing solely on this entity. Proper indications for this procedure and eligible selection criteria are urgently required considering the risks, associated with this approach, especially when myeloablative conditioning is being performed [16, 20]. Though some studies suggested the development of a graft *versus* HL effect [19–21, 25, 30, 32, 33], its existence remains still unproven. As the donor type does not seem to have a dramatic impact on the outcomes, the intensity of conditioning represents the most important transplant-associated parameter in this term. The introduction of RIC in the transplant setting improved NRM rates, but the incidence of relapse was high. Therefore, the question about an optimal intensity of conditioning in relapsed/refractory HL patients remains open. Heavily pretreated patients and patients with comorbidities should preferably undergo reduced conditioning, whereas some younger patients might have a benefit from myeloablative (e.g., BuCy) or less intensified conventional regimens (e.g., BEAM) due to the lower relapse rates [22, 31]. The use of *in vivo* T-cell depletion might contribute to better survival outcomes [33]. Though the present role of the allogeneic SCT in relapsed/refractory HL patients is not defined, some retrospective studies have already shown a realistic clinical benefit of this approach as compared to conventional chemotherapy [28]. In this setting, prospective multicenter study are needed to standardize the indication and the time point of allogeneic SCT in therapeutic algorithms for patients with relapsed or refractory Hodgkin lymphoma.

## Figures and Tables

**Table 1:** Allogeneic SCT for relapsed/refractory HL patients (HL: Hodgkin's Lymphoma; no.: number; DLIs: donor lymphocyte infusions; TRM: transplant-related mortality; DFS: disease-free survival; PFS: progression-free survival; GvL: graft *versus* lymphoma effect; FM(-A): fludarabine/melphalan (+alemtuzumab); MAC: myeloablative conditioning regimens; RIC: reduced intensity conditioning regimens; UD: unrelated donor; MRD: matched related donor; HD: haploidentical donor; NS: not stated).

Reference	No. of transplant recipients	Patients with chemoresistant HL (no.)	Early TRM	Cumulative TRM (-year)	Relapse (-year)	DFS/PFS (-year)	Response to immuno-therapy with DLIs	GvL effect
Gajewski et al. [16]	100	89	13%	61% (3)	65% (3)	15% (3)	NS	No

Milpied et al. [19]	45	NS	31%	48% (4)	61% (4)	15% (4)	NS	Possible

Anderson et al. [20]	53	NS	NS	49% (5)	65% (5)	18% (5)	NS	Possible

Akpek et al. [21]	53	28	24% (chemosensitive)	32% (chemosensitive)	53% (10)	26% (10)	NS	Possible
			39% (chemoresistant)	53% (chemoresistant)				

Anderlini et al. [26]	40	14	5%	22% (1.5)	55% (1.5)	32% (1.5)	3/8 (38%)	No

Devetten et al. [29]	143	67	15%	33% (2)	47% (2)	20% (2)	NS	NS

Burroughs et al. [30]	90	16 (MRD)	16% (MRD)	21% (2) (MRD)	56% (2) (MRD)	23% (2) (MRD)	NS	Possible
		8 (UD)	0% (UD)	8% (2) (UD)	63% (2) (UD)	29% (2) (UD)		
		6 (HD)	0% (HD)	9% (HD)	40% (2) (HD)	51% (2) (HD)		

Robinson et al. [25]	285	72	11%	21% (3)	59% (5)	25% (3)	41/79 (52%)	Possible

Claviez et al. [31]	91	32	NS	26% (5)	44% (5)	30% (5)	2/12 (17%)	No

Anderlini et al. [27]	58	28	7%	15% (2)	55% (2)	32% (2)	6/14 (43%)	No

Alvarez et al. [32]	40	20	13%	25% (1)	NS	32% (2)	6/11 (55%)	Possible

Peggs et al. [33]	67	10 (FM-A)	NS	7% (2) (FM-A)	54% (3) (FM-A)	43% (4) (FM-A)	13/19 (68%) (FM-A)	Possible
		19 (FM)		29% (2) (FM)	44% (3) (FM)	25% (4) (FM)	6/11 (55%) (FM)	

Sureda et al. [34]	168	43 (MAC)	28% (MAC)	48% (3) (MAC)	30% (3) (MAC)	20% (MAC)	NS	Possible
		49 (RIC)	15% (RIC)	24% (3) (RIC)	57% (3) (RIC)	18% (RIC)		

Sarina et al. [28]	104	36	NS	13% (2)	54% (2)	31% (2)	NS	Possible
